# 

**Published:** 1892-11-05

**Authors:** 


					WITH EXTRA NURSING SUPPLEMENT
Per Annum, 8s. 6d.t post free,
Monthly Farts, 6d.; post free, 8d.
Ditto per Annum, 8s., post free.
No. 319. Vol. XIII.] Saturday,
November 5th, 1892.
rTwOPENOK.

				

